# An estrogen receptor (ER)‐related signature in predicting prognosis of ER‐positive breast cancer following endocrine treatment

**DOI:** 10.1111/jcmm.14338

**Published:** 2019-05-23

**Authors:** Jianing Tang, Qiuxia Cui, Dan Zhang, Xing Liao, Jian Zhu, Gaosong Wu

**Affiliations:** ^1^ Department of Thyroid and Breast Surgery Zhongnan Hospital of Wuhan University Wuhan China; ^2^ Department of Thyroid and Breast Surgery Tongji Hospital, Huazhong University of Science and Technology Wuhan China

**Keywords:** breast cancer, estrogen receptor, nomogram, prognosis

## Abstract

Quite a few estrogen receptor (ER)‐positive breast cancer patients receiving endocrine therapy are at risk of disease recurrence and death. ER‐related genes are involved in the progression and chemoresistance of breast cancer. In this study, we identified an ER‐related gene signature that can predict the prognosis of ER‐positive breast cancer patient receiving endocrine therapy. We collected RNA expression profiling from Gene Expression Omnibus database. An ER‐related signature was developed to separate patients into high‐risk and low‐risk groups. Patients in the low‐risk group had significantly better survival than those in the high‐risk group. ROC analysis indicated that this signature exhibited good diagnostic efficiency for the 1‐, 3‐ and 5‐year disease‐relapse events. Moreover, multivariate Cox regression analysis demonstrated that the ER‐related signature was an independent risk factor when adjusting for several clinical signatures. The prognostic value of this signature was validated in the validation sets. In addition, a nomogram was built and the calibration plots analysis indicated the good performance of this nomogram. In conclusion, combining with ER status, our results demonstrated that the ER‐related prognostic signature is a promising method for predicting the prognosis of ER‐positive breast cancer patients receiving endocrine therapy.

## 1 INTRODUCTION

Breast cancer is a heterogeneous disease with multiple molecular features. It is a major health burden in the world, which results in the leading cause of cancer death among females. Incidence rate of breast cancer has been increased for several years, resulting from a combination of social and economic factors, including the postponement of childbearing, obesity and physical inactivity.^1^ Molecular studies have demonstrated that there were at least four molecular subtypes of breast cancer: luminal, basal, human epidermal growth factor receptor 2 (HER2)‐enriched and normal‐like. These subtypes exhibit different histopathological features and treatment sensitivities.^2^

Luminal A and luminal B are the most two common subtypes of breast cancer, which accounts for approximately 70% of all cases. They are characterized by the expression of estrogen receptor (ER) and progesterone receptor (PR). ER‐related genes are highly expressed in luminal A tumours, while expression levels of HER2 and some proliferation‐related genes are low. Compared with luminal A tumours, luminal B tumours have lower expression levels of ER‐related genes, higher expression of the proliferation‐related genes and variable expression of HER2 genes. Patients with luminal A breast cancer were often considered to have the best prognosis, followed by patients with luminal B breast cancer.^3^ Expression of ER is associated with favourable prognosis and can predict the efficacy of endocrine therapies including aromatase inhibitors and tamoxifen. Previous studies demonstrated that ER‐positive breast cancer patients treated with adjuvant tamoxifen treatment resulted in a decreased breast cancer death. Despite most ER‐positive breast cancer patients show good prognosis after receiving antiestrogen therapy, while some of them can develop acquired resistance after 5 years of therapy and suffer from distant metastasis or even death.^4^

The high‐throughput platforms for genomic analysis provided promising tools in medical oncology with great clinical applications. Multiple gene prognostic signatures could provide further prognostic information, and several molecular prognostic profiles have been validated and are in clinical use: the Oncotype Dx, the Amsterdam 70‐gene signature and the PAM50 are the three most commonly used. The Oncotype DX calculates a recurrence score and divides breast tumours into low‐, intermediate‐ and high‐risk groups to estimate the likelihood of recurrence in tamoxifen‐treated patients with (ER)‐positive breast cancer.^5^, ^6^ The Amsterdam 70‐gene signature could accurately grouped patients into low or high risks to predict distant metastases and death, which is approved for application in both ER‐positive and ER‐negative tumours.^7^ The PAM50 is a 50‐gene test, improving classification of breast cancer patients into prognostic groups.^8^ These signatures assist therapeutic strategies determination and prognosis predication of patients with breast cancer.

Expression of ER‐related genes could provide predictive value for predicting the responses to chemotherapy, and may allow to identify patients who will either benefit or be resistant to chemotherapy.^9^ In this study, we constructed an ER‐related gene signature and developed a nomogram to predict the relapse‐free survival (RFS) of ER‐positive breast cancer patients receiving endocrine therapy. Our findings suggested that this ER‐related gene signature could be used as an effective prognostic predictor for patients with ER‐positive breast cancer patients receiving endocrine therapy.

## 2 MATERIALS AND METHODS

### 2.1 Data processing

Three datasets (GSE6532, GSE4922 and GSE9195) containing gene expression profiling data of ER‐positive breast cancer patients receiving adjuvant hormonal therapy alone and their corresponding clinical data were downloaded from the GEO databases. Only ER‐positive patients with complete clinical information were included in our analysis. Three chip platforms, Affymetrix Human Genome U133A (GPL96), Affymetrix Human Genome U133B (GPL97) and Affymetrix Human Genome Plus 2.0 (GPL570) were used to obtain gene expression data. Raw microarray cell intensity files were obtained, background‐adjusted and normalized using Robust Multichip Average. The RNA expression data were scaled with a standard deviation of 1 and a mean of 0. The data under the same chip platform were then merged and the ComBat method was used to remove the potential internal and external batch effects. We reannotated the probe sets of the Affymetrix Human Genome U133A, Affymetrix Human Genome U133B and Affymetrix Human Genome Plus 2.0 platforms by mapping all probes to the Gencode annotation (Version 29) using SeqMap. We selected the probes that were mapped uniquely to the genome with no mismatch. We obtained 256 ER‐related genes through the Molecular Signature Database v6.2 2 3. Only 62 genes mapped to all the three platforms were used for further analysis.

### 2.2 Construction of the ER‐related prognostic signature

The dataset based on GPL96 was used as the training set, and another two sets based on GPL97 and GPL570 were used as validation sets. GSE12093 and GSE17705 based on platform GPL96 containing survival information were also downloaded and combined for validation. Univariate Cox regression analysis was first performed to identify prognostic genes. *P* < 0.05 was considered as significant. Lasso‐penalized Cox regression was used to further narrow the genes for prediction of the RFS. The LASSO Cox regression model was analysed using the ‘glmnet' package. LASSO shrinked all regression coefficients towards 0 and set the coefficients of many irrelevant features exactly to 0 based on the regulation weight λ. The optimal λ was selected according to minimum cross validation error in 10‐fold cross validation. Finally, a multivariate Cox regression analysis was conducted to assess the contribution of genes as an independent prognostic factor for patient survival. A stepwise method was employed to select the best model. A risk score was built, with the coefficients weighted by the penalized Cox model in the training set. The optimal cut‐off of risk score was obtained using ‘survminer' package in r. All patients were classified into either the high‐risk or the low‐risk group based on the optimal cut‐off of risk score.

### 2.3 Construction of the nomogram

A nomogram was constructed using the ‘rms' r package and calibration plots were performed to assess the prognostic accuracy of the nomogram. The predicted outcomes and observed outcomes of the nomogram were presented in the calibrate curve and the 45° line represented the best prediction.

### 2.4 Statistical analysis

To investigate the prognostic accuracy of ER‐related classifier, we performed time‐dependent receiver operating characteristic (ROC) analysis using the ‘survivalROC' R package. Relapse‐free survival was analysed based on Kaplan‐Meier method, and we performed the log‐rank test to assess the statistical significance of the differences between different groups. Cox regression model was used to analyse multivariable survival analysis. Hazard ratios (HRs) with their respective 95% confidence intervals were obtained. A *P* < 0.05 was considered statistically significant and all tests were two‐sided. All statistical tests were performed with r software (version 3.5.0).

## 3 RESULTS

### 3.1 Patient characteristics

**Figure 1:**
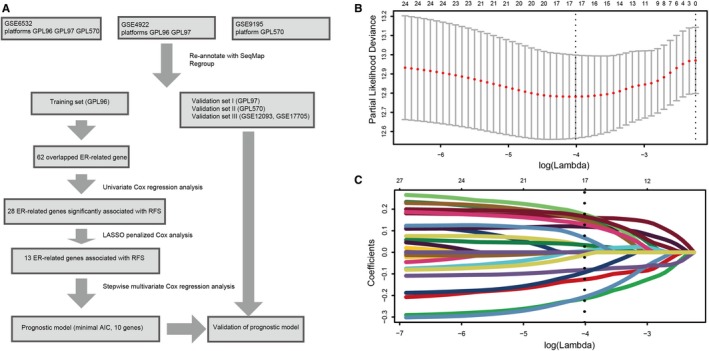
Flow chart and 10‐fold cross‐validation for tuning parameter selection. A, Flow chart indicating the process used to select target genes included in the analysis. B, 10‐fold cross‐validation for tuning parameter selection in the Lasso model. C, LASSO coefficient profiles of the 28 prognostic genes. A vertical line is drawn at the value chosen by 10‐fold cross‐validation

As shown in Figure 1, a flow chart of the analysis procedure was developed to describe our study. We collected breast cancer expression datasets and their corresponding clinical data from GEO database. Cases from GSE GSE6532, GSE4922 and GSE9195 were assigned to three sets: training set (GPL96), validation set I (GPL97) and validation set II (GPL570). Clinical information included age, tumour size, grade and lymph node status. As GSE12093 and GSE17705 did not have the information of age, tumour size, these two datasets containing 434 ER‐positive patients were combined for validation (validation set III).

The clinicopathologic characteristics of patients in the training set are shown in Table 1. The median follow‐up in training set was 9.5 years (low‐risk group) and 5.2 years (high‐risk group); in the validation set I, median follow‐up was 8.9 years (low‐risk group) and 4.9 years (high‐risk group); in the validation set II, median follow‐up was 10.5 years (low‐risk group) and 7.2 years (high‐risk group); in the validation set III, median follow‐up was 8.7 years (low‐risk group) and 6.9 years (high‐risk group). Ninety‐three (50%, training set), 92 (54.1%, validation set I), 25 (39.1%, validation set II) and 74 (28.7%, validation set III) patients in high‐risk group developed relapse during the follow‐up period.

**Table 1:** Clinicopathologic characteristics of three sets of breast cancer patients according to the ER‐related signature

Variables	Training set GPL96 (n = 357)		Validation set I GPL97 (n = 421)		Validation set II GPL570 (n = 128)		Validation set III GPL96 (n = 434)	
	Low risk (%)	High risk (%)	Low risk (%)	High risk (%)	Low risk (%)	High risk (%)	Low risk (%)	High risk (%)
Age at diagnosis (y)								
Median	59	63	62	61	63	64		
≤50	44 (25.7)	37 (19.9)	54 (21.5)	36 (21.2)	6 (9.4)	3 (4.7)		
>50	127 (74.3)	149 (80.1)	197 (78.5)	134 (78.8)	58 (90.6)	61 (95.3)		
Tumour size								
≤2 cm	135 (78.9)	129 (69.4)	160 (63.7)	76 (44.7)	35 (54.7)	25 (39.1)		
>2 cm	36 (21.1)	57 (30.6)	91 (36.3)	94 (55.3)	29 (45.3)	39 (60.9)		
Lymph node status								
Negative	131 (76.6)	111 (59.7)	185 (73.7)	92 (54.1)	26 (40.6)	28 (43.8)		
Positive	40 (23.4)	75 (40.3)	66 (26.3)	78 (45.9)	38 (59.4)	36 (56.3)		
Grade								
I	63 (36.8)	26 (14.0)	86 (34.3)	35 (20.6)	25 (39.1)	6 (9.4)		
II	101 (59.1)	127 (68.3)	136 (54.2)	94 (55.3)	22 (34.4)	35 (54.7)		
III	7 (4.1)	33 (17.7)	29 (11.6)	41 (24.1)	17 (26.6)	23 (35.9)		
Disease‐relapse event	33 (19.3)	93 (50.0)	55 (21.9)	92 (54.1)	7 (10.9)	25 (39.1)	17 (9.6)	74 (28.7)
Median follow‐up (y)	9.5	5.2	8.9	4.9	10.5	7.2	8.7	6.9

### 3.2 Identification of an ER‐related signature

**Figure 2:**
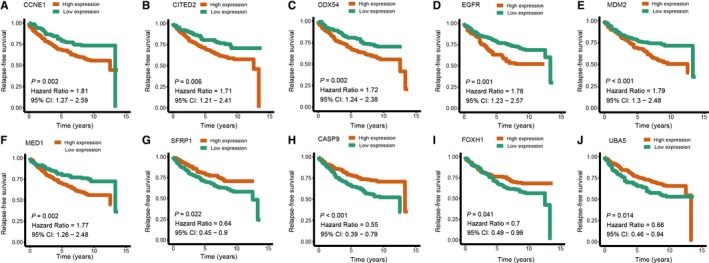
Univariate Cox regression analysis of the ten prognostic genes in the signature. A, CCNE1. B, CITED2. C, DDX54. D, EGFR. E, MDM2. F, MED1. G, SFRP1. H, CASP9. I, FOXH1. J, UBA5

We first performed univariate Cox regression analysis to identify prognostic genes in the training set. The patients were stratified into high expression group and low expression group according to optimal cut‐off of each gene. And 28 ER‐related genes significantly associated with the RFS were considered as prognostic genes and selected for further analysis. Then Lasso‐penalized Cox analysis with 10‐fold cross‐validation was performed to narrow the genes for prediction of the RFS, 13 ER‐related genes were identified. Subsequently, we conducted a stepwise multivariate Cox regression analysis, and 10 ER‐related genes were finally screened out as prognostic genes to build a predictive model. The predictive model was defined as the linear combination of the expression levels of the 10 ER‐related genes weighted by their relative coefficient in the multivariate Cox regression model, as risk score = (0.24 × expression of CCNE1) + (0.19 × expression of CITED2) + (0.32 × expression of DDX54) + (0.16 × expression of EGFR) + (0.26 × expression of MDM2) + (0.23 × expression of MED1) + (−0.47 × expression of SFRP1) + (−0.23 × expression of CASP9) + (−0.26 × expression of FOXH1) + (−0.22 × expression of UBA5). Among the 10 prognostic genes, CCCNE1, CITED2, DDX54, EGFR, MDM2 and MED1 showed positive coefficients in the Cox regression analysis, indicating that their high expression signified a shorter RFS. SFRP1, CASP9, FOXH1 and UBA5 showed negative coefficients, suggesting their high expression was associated with better RFS. These results were consistent with the previous univariate Cox regression analysis (Figure 2).

### 3.3 Analysis of the ER‐related signature in the training set

**Figure 3:**
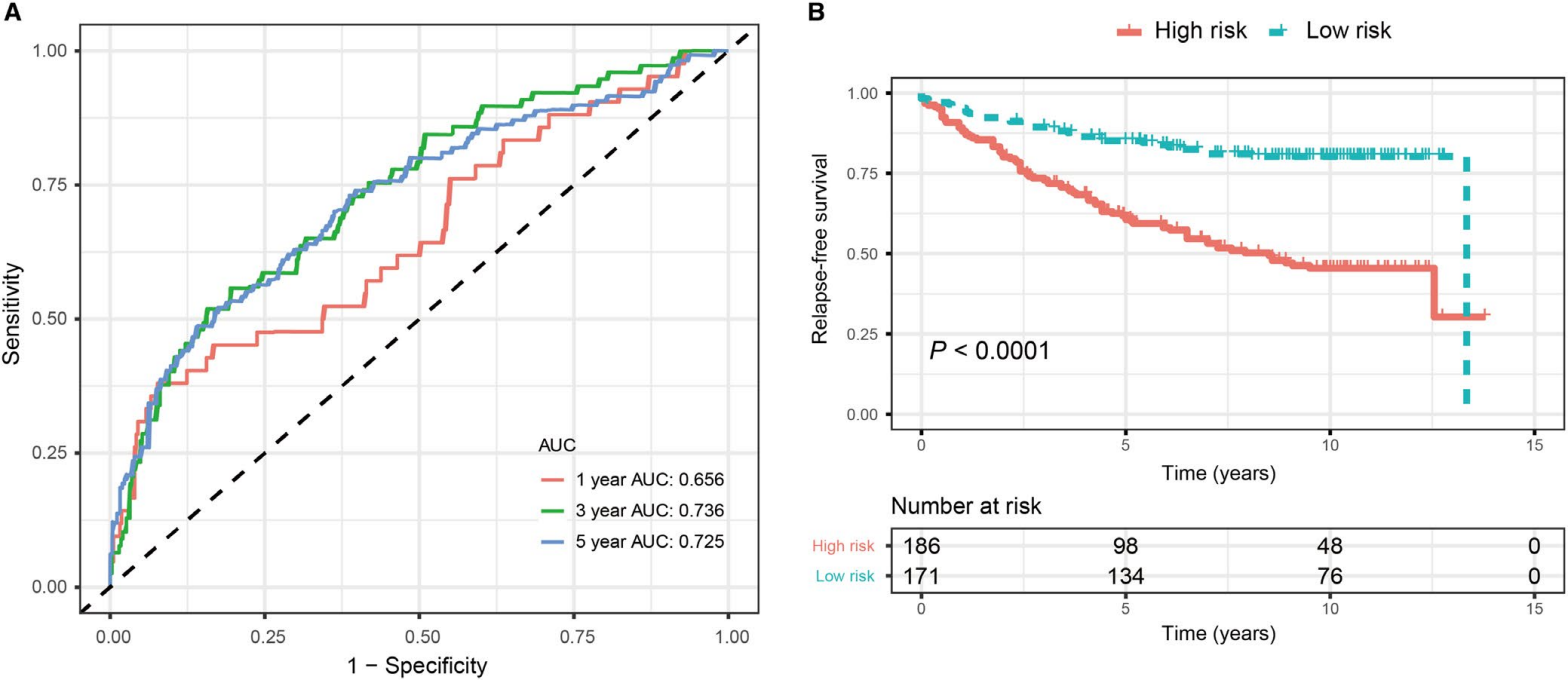
Validation of prognostic risk score model in training set. A, Time‐dependent ROC curves of the ER‐related signature. B, Kaplan‐Meier survival analysis of the ER‐related signature

**Figure 4:**
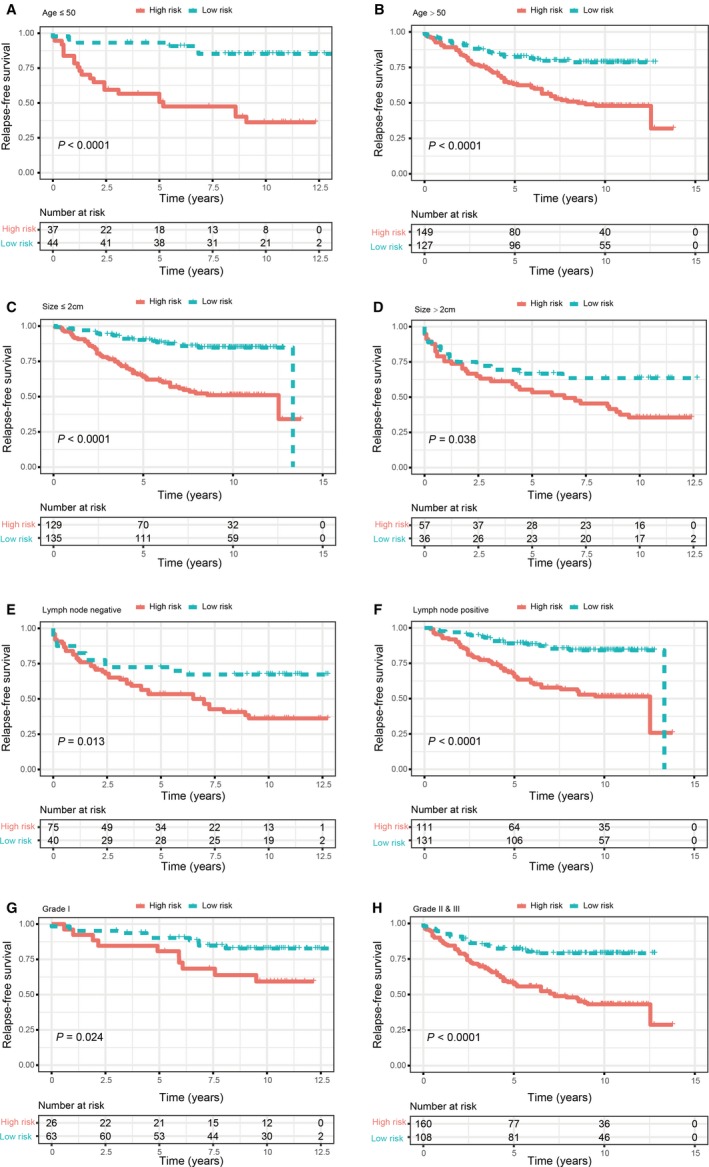
Kaplan‐Meier survival analysis for patients according to the ER‐related‐based signature stratified by clinicopathological risk factors. (A,B). Age. (C,D). Tumour size. (E,F). Lymph node status. (G,H). Tumour grade

In the training set, we performed the time‐dependent ROC curves analysis to assess the prognostic accuracy of the ER‐related signature. The areas under the ROC curve (AUC) achieved 0.656, 0.736 and 0.735 at 1, 3 and 5 years of recurrence‐free survival (Figure 3A). The risk score for each patient was calculated and we classified all breast cancer patients in the training set into high‐risk group and low‐risk group by using the optimum cut‐off score (0.1) generated by ‘survminer' package in R via the maximally selected rank statistics. We found that patients in the lower‐risk group had significantly better RFS than those in high‐risk group (Figure 3B). Multivariate Cox proportional hazards regression analysis demonstrated that the ER‐related signature was an independent risk factor when adjusting for the classical clinicopathologic factors (Table 2). When we stratified the patients by clinicopathological risk factors, the ER‐related signature was still a statistically significant prognostic model where patients in high‐risk group had poorer prognosis (Figure 4). The same results were found in the entire validation set (Figure S1).

**Table 2:** Multivariate Cox proportional hazards regression analysis of the clinicopathologic characteristics and the ER‐related signature with RFS

Variable	Training set		Validation set I		Validation set II	
	HR (95% CI)	*P*	HR (95% CI)	*P*	HR (95% CI)	*P*
Age (≦50 vs >50 y)	0.802 (0.520, 1.236)	0.317	0.635 (0.429, 0.941)	0.024	1.005 (0.965, 1.046)	0.816
Tumour size (≦2 cm vs >2 cm)	1.618 (1.092, 2.398)	0.016	2.047 (1.434, 2.922)	<0.001	1.551 (0.97, 3.452)	0.282
Lymph node status (Negative vs Positive)	1.571 (1.081, 2.282)	0.018	1.300 (0.914, 1.848)	0.144	1.275 (0.578, 2.814)	0.547
Tumor grade (Grade I vs Grade II & III)	1.276 (0.777, 2.094)	0.336	1.430 (0.935, 2.187)	0.099	3.366 (0.774, 14.638)	0.106
Integrated RNA signature (low risk vs high risk)	2.758 (1.815, 4.189)	<0.001	2.653 (1.883, 3.739)	<0.001	3.864 (1.632, 9.151)	0.002

### 3.4 Validation of the ER‐related signature in validation sets

**Figure 5:**
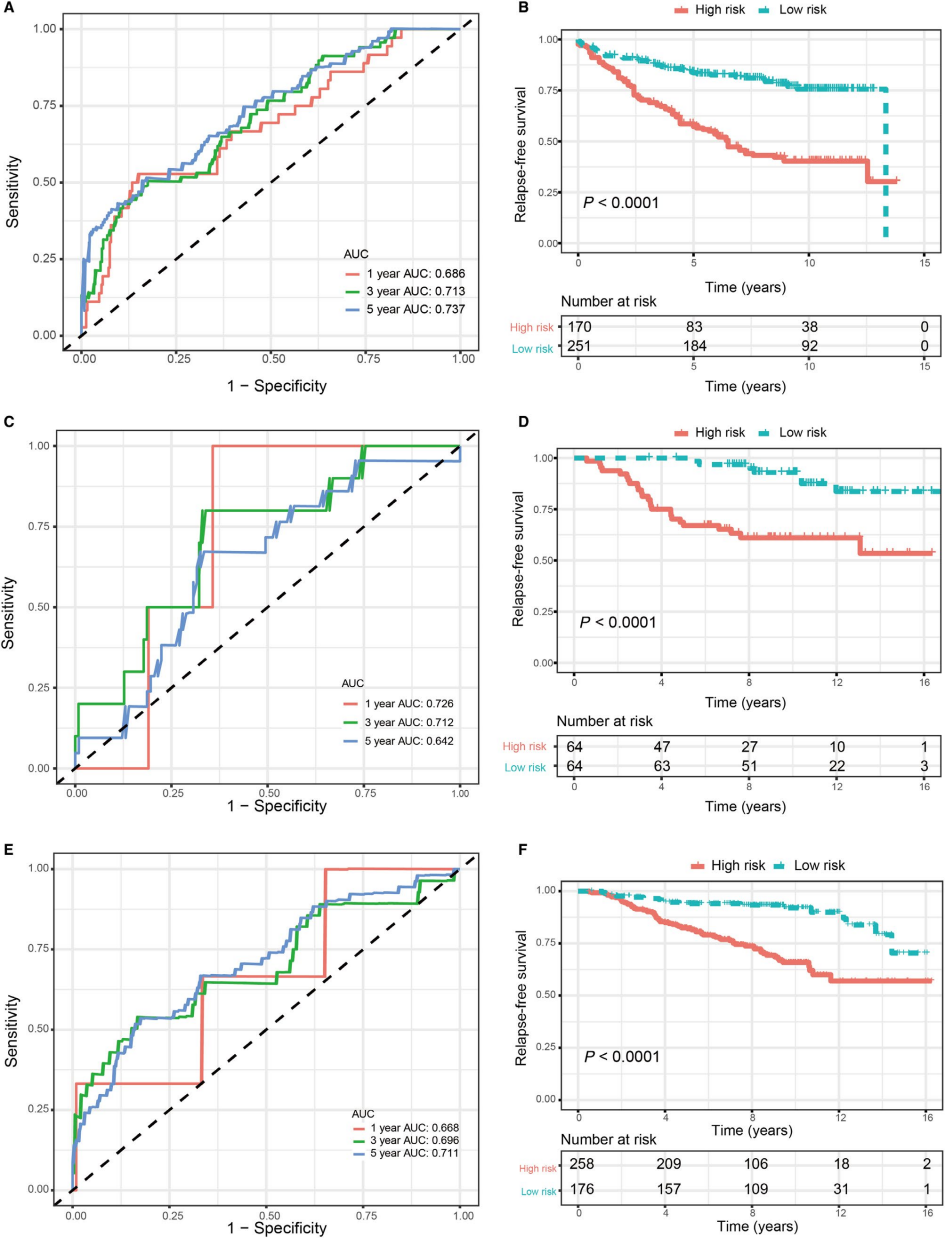
Validation of ER‐related signature in validation sets. A, Time‐dependent ROC curves of the ER‐related signature in validation set I. B, Kaplan‐Meier survival analysis of the ER‐related signature in validation set I. C, Time‐dependent ROC curves of the ER‐related signature in validation set II. D, Kaplan‐Meier survival analysis of the ER‐related signature in validation set II. E, Time‐dependent ROC curves of the ER‐related signature in validation set III. F, Kaplan‐Meier survival analysis of the ER‐related signature in validation set III

To validate the predictive power of the ER‐related signature for breast cancer patients, we tested the signature in three validation sets. According to the signature identified above, patients in the lower‐risk group had significantly better RFS In validation set I, AUCs at 1, 3 and 5 years were 0.686, 0.713 and 0.737. In validation set II, AUCs at 1, 3 and 5 years were 0.726, 0.712 and 0.642. In validation set III, AUCs at 1, 3 and 5 years were 0.668, 0.696 and 0.711. In the three validation sets, Kaplan‐Meier analysis and log‐rank test demonstrated that patients in the lower‐risk group had significantly better RFS (Figure 5). Multivariate Cox proportional hazards regression analysis of validation set I and II also demonstrated that the signature was an independent risk factor (Table 2).

### 3.5 Nomogram development

**Figure 6:**
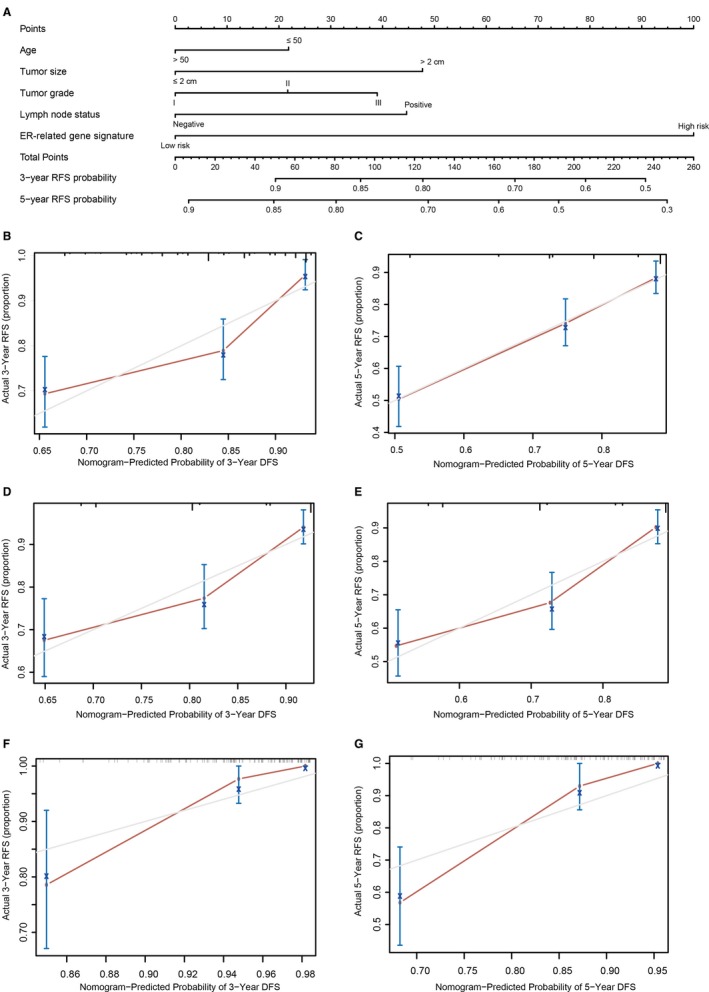
Nomogram to predict risk of cancer recurrence. A, Nomograms to predict risk of cancer recurrence. B, 3‐year nomogram calibration curves of training set. C, 5‐year nomogram calibration curves of training set. D, 3‐year nomogram calibration curves of validation set I. E, 5‐year nomogram calibration curves of validation set I. F, 3‐year nomogram calibration curves of validation set II. G, 5‐year nomogram calibration curves of validation set II

To predict the recurrence probability of breast cancer patients using a quantitative method, we constructed a nomogram that integrated both the ER‐related signature and the conventional clinicopathological factors (Figure 6) to predict 3‐ and 5‐year DFS probability. Calibration plots indicated that the nomograms had good accuracy compared with an ideal model both in training set and validation set (Figure 6B‐G).

## 4 DISCUSSION

Recent advances of high‐throughput technologies for genomic analysis provide promising tools in medical oncology with great clinical applications. However, it is difficult to use such a large number of genes for clinical application. The ER‐positive breast cancer is the most common molecular subtype of breast cancer. Patients with ER‐positive breast cancer are often treated with endocrine therapy and are associated with a favourable prognosis. ER‐related genes and pathways are involved in the progression and chemoresistance of breast cancer. It is a novel and important method that combining ER status with ER‐related genes for predicting the response of endocrine therapy and prognosis of breast cancer patients.

In our present study, using high‐throughput expression profiling, we constructed and validated an ER‐related gene signature (CCNE1, CITED2, DDX54, EGFR, MDM2, MED1, SFRP1, CASP9, FOXH1 and UBA5) to predict RFS for ER‐positive breast cancer patients. The training set based on GPL96 platform was used to identify the signature. After univariate, Lasso and multivariate Cox analysis, 10 genes were selected to construct a multi‐gene signature for prognosis prediction. The ER‐related gene signature was first assessed in the training set. Patients in low‐risk group had significantly better survival than those in high‐risk group. Results of ROC analysis showed that this signature exhibited excellent diagnostic efficiency for the 1‐, 3‐ and 5‐year disease‐relapse event. Moreover, multivariate Cox regression model indicated this ER‐related gene signature as an independent risk factor when adjusting for several clinical features such as age, tumour grade, tumour size and lymph node status. When patients were stratified by clinicopathological features, the ER‐related gene signature remained a strong prognostic model. Similar results were observed when further validated in validation sets. These results demonstrated that this ER‐related gene signature could successfully categorize patients into high‐risk and low‐risk groups with different RFS and was an effective prognostic indicator for breast cancer patients. In addition, a nomogram was developed that integrated both the ER‐related gene signature and clinicopathological risk factors and to accurately predict the likelihood of RFS in patients with breast cancer. Calibration plots indicated that the actual RFS corresponded closely with predicted RFS, suggesting our nomograms had good predictive performance both in training and validation sets.

The biological functions of these ER‐related genes have been previously studied. CCNE1 encodes a protein which belongs to the cyclin family, whose members are characterized by a dramatic periodicity in protein abundance through the cell cycle and plays a critical role in promoting cell‐cycle progression. Several studies have demonstrated that up‐regulation of CCNE1 is associated with higher tumour grades and poor prognosis in many tumours.^10^, ^11^, ^12^, ^13^ In breast cancer, CCNE1 is the immediate downstream effector of estrogen‐related receptor α.^14^ Overexpression of cyclin E contributes to the antiestrogen resistance.^15^ And up‐regulation of CCNE1 can abrogate the tamoxifen‐mediated growth arrest via the modification of RB/E2F pathway.^14^ CITED2 has been reported to play a role in tumourigenesis, including that of the colon, lung and skin.^16^, ^17^, ^18^ In a murine mammary cancer model, CITED2 is identified as a potential facilitator of breast cancer bone metastasis.^19^ Compared with primary tumours, expression of CITED2 is significantly up‐regulated in metastatic lesions, with the highest levels in bone metastasis.^20^ Overexpression of CITED2 could cause tamoxifen resistance in breast cancer cell lines.^21^ And studies demonstrated that CITED2 can elevate ER transcriptional activity, thus reducing the response to antiestrogen therapy.^22^ DDX54 acts as a corepressor of the ligand ER, which can dampen stimulation and intensify repression of estradiol‐ER‐regulated genes.^23^ Accumulating studies demonstrated that EGFR overexpression is associated with poor prognosis of breast cancer, and targeting EGFR therapy can enhance the sensitivity of breast cancer cells to chemotherapy.^24^, ^25^, ^26^ EGF can promote phosphorylation of serine and tyrosine residues in ER, and direct interaction is observed between ER and EGFR. Communication between EGFR and ER can enhance proliferation and reduce the apoptosis of breast cancer cells.^27^ MDM2 is overexpressed in a variety of malignancies, including sarcomas, leukaemias and solid tumours, which plays a crucial role in the development and progression of tumour. Overexpression of MDM2 is associated with drug resistance and poor clinical prognosis.^28^, ^29^ Chromatin immunoprecipitation assay has indicated the recruitment of ER to the MDM2 promoter, suggesting the regulatory role of ER in the MDM2 expression.^30^ MDM2 can also regulate ER stability and transcriptional activity in human cancer cells.^31^ ER coactivator protein MED1 is reported as a novel biomarker which plays role in co‐activating ER and results in tamoxifen resistance.^32^, ^33^ SFRP genes are antagonists of Wnt pathway, and they are potential tumour suppressors in gastric, colon, ovarian, lung and breast cancers.^34^ Compared with ER‐negative breast cancers, expression of SFRP1 is reduced in ER‐positive breast cancers. Loss of SFRP1 may lead to enhanced estrogen‐mediated proliferation.^35^ In the mitochondrial cell death pathway, the CASP9 protein acts as an initiator caspase of apoptosis, up‐regulation of CASP9 reduces the viability of breast cancer cells due to apoptosis induction.^36^ FOXH1 has been found to inhibit the transcriptional activities of ER, knockdown of FOXH1 increased estrogen‐dependent cell growth in breast cancer cells.^37^ UBA5 plays a crucial role in involved in ASC1 ufmylation, which is required for ERα transactivation and tumour formation.^38^ The biological roles of these ER‐related genes remain largely unclear in breast cancer, further studies are required to investigate the underlying molecular mechanisms.

In this study, we developed a prognostic signature based on 10 ER‐related genes and constructed a novel nomogram to predict the RFS. These findings might lead to the development of a cheap molecular test and suitable in the clinical routine. Although the nomogram demonstrated an accurate survival prediction, several limitations should not be ignored. The sample size of our study was limited, and large‐scale cohort studies are performed to investigate the prognostic value of this ER‐related signature. As only the patients who had complete information were included in present study, there might be a selection bias in the primary cohort. Several predictors, such as radiotherapy and Ki‐67 index, were not analysed. Further, in vivo and in vitro studies are required to confirm the exact molecular mechanisms of these diagnostic genes.

In conclusion, combining with ER status, our results demonstrated that the ER‐related prognostic signature is a novel and important method for predicting the prognosis of breast cancer patients. Thereby, it may be a useful predictive tool with a good prospect of clinical application for ER‐positive breast cancer patients receiving endocrine therapy.

## CONFLICT OF INTEREST

The authors declare that they have no conflict of interest.

## AUTHOR CONTRIBUTIONS

JT, JZ and GW reviewed relevant literature and drafted the manuscript. DZ, XL and QC conducted all statistical analyses. All authors read and approved the final manuscript.
