# Supplementary figures and images for: An estrogen receptor (ER)‐related signature in predicting prognosis of ER‐positive breast cancer following endocrine treatment
Source: J Cell Mol Med. 2019 May 23;23(8):4980–90. doi: 10.1111/jcmm.14338 (PMC6652714; doi:10.1111/jcmm.14338)

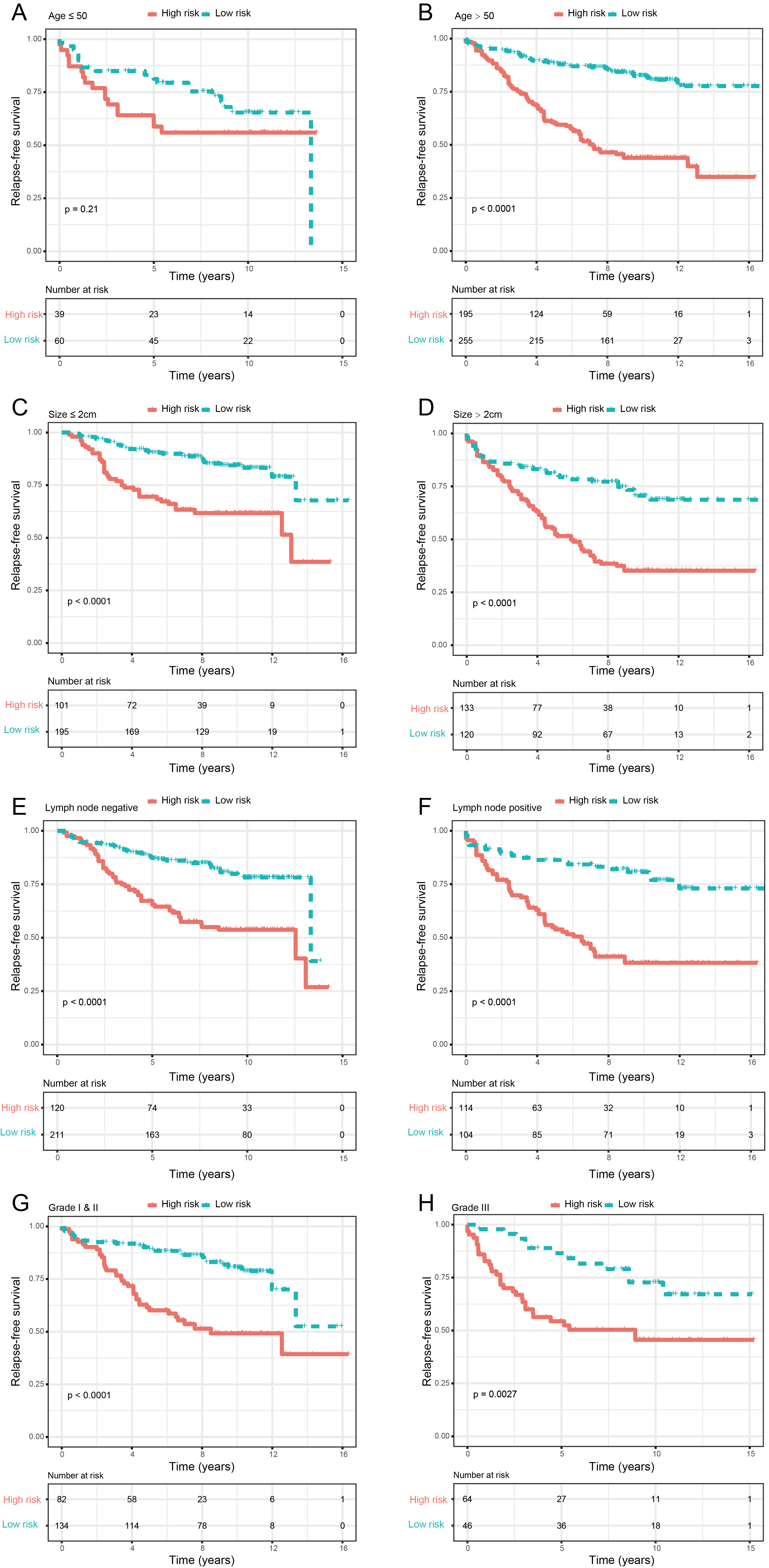

Supplement: Supplementary file 1 [file JCMM-23-4980-s001.tif]
