# Supplementary material for: An estrogen receptor (ER)‐related signature in predicting prognosis of ER‐positive breast cancer following endocrine treatment
Source: J Cell Mol Med. 2019 May 23;23(8):4980–90. doi: 10.1111/jcmm.14338 (PMC6652714; doi:10.1111/jcmm.14338)
Supplement: Supplementary file 2 [file JCMM-23-4980-s002.docx]

**Figure S1: Kaplan-Meier survival analysis for patients according to the ER-related-based signature stratified by clinicopathological risk factors in validation set.** (A, B). Age. (C, D). Tumor size. (E, F). Lymph node status. (G, H). Tumor grade
